# Aboriginal and Torres Strait Islander Complex Trauma and Strengths Questionnaire: psychometric evaluation

**DOI:** 10.1080/00049530.2024.2335917

**Published:** 2024-05-23

**Authors:** Graham Gee, Tess Bright, Amy Morgan, Carlie Atkinson, Shawana Andrews, Yvonne Clark, Karen Glover, Tanja Hirvonen, Elise Davis, Kimberley A. Jones, Rachel Reilly, Fiona Mensah, Madelyne Hudson-Buhagiar, Shannon K Bennetts, Helen Herrman, Helen Milroy, Andrew Mackinnon, Catherine Chamberlain

**Affiliations:** a Intergenerational Health Group, Murdoch Children’s Research Institute, Parkville, Victoria, Australia; b School of Psychological Sciences, University of Melbourne, Parkville, Victoria, Australia; c Indigenous Health Equity Unit, Onemda: Aboriginal and Torres Strait Islander Health and Wellbeing, Melbourne School of Population and Global Health, University of Melbourne, Melbourne, Victoria, Australia; d Centre for Mental Health, Melbourne School of Population and Global Health, University of Melbourne, Parkville, Victoria, Australia; e We Al-Li Pty Ltd, Goolmangar, NSW, Australia; f Melbourne Poche Centre for Indigenous Health, University of Melbourne, Parkville, Victoria, Australia; g South Australian Health and Medical Research Institute (SAHMRI), Adelaide, South Australia, Australia; h UniSA Justice and Society, University of South Australia, Magill, South Australia, Australia; i Centre of Best Practice in Aboriginal and Torres Strait Islander Suicide Prevention, School of Indigenous Studies, University of Western Australia, Perth, WA, Australia; j College of Medicine and Public Health, Flinders University, Adelaide, SA, Australia; k Department of Paediatrics, University of Melbourne, Parkville, Victoria, Australia; l Wurru Wurru Health Unit, University of Melbourne, Parkville, Victoria, Australia; m Judith Lumley Centre, School of Nursing and Midwifery, La Trobe University, Bundoora, Victoria, Australia; n Orygen, and Centre for Youth Mental Health, University of Melbourne, Parkville, Victoria, Australia; o Perth Children’s Hospital and University of Western Australia, Division of Psychiatry, Faculty of Health and Medical Sciences, Perth, WA, Australia; p Black Dog Institute, University of New South Wales, New South Wales, Australia; q Ngangk Yira: Research Centre for Aboriginal Health and Social Equity, Murdoch University, Perth, Western Australia, Australia

**Keywords:** Aboriginal and Torres Strait Islander, Indigenous, complex trauma, complex post-traumatic stress disorder, parenting, exploratory factor analysis

## Abstract

**Objective:**

Complex post-traumatic stress disorder (complex trauma) describes a cluster of symptoms frequently associated with prolonged exposure to inescapable threats or abuse. For Aboriginal and Torres Strait Islander peoples in Australia impacted by complex trauma, there may be compounding factors, such as experiences of historical trauma, loss and socio-economic deprivation stemming from colonisation. However, there is no culturally appropriate tool to assess complex trauma. This paper presents the psychometric evaluation of a preliminary version Aboriginal and Torres Strait Islander Complex Trauma and Strengths Questionnaire (ACTSQ).

**Methods:**

Following 2 years of rigorous Aboriginal-led co-design, participants were recruited through community networks and partner health services in South Australia, Victoria, and Northern Territory (October 2020–May 2022). A trained interviewer contacted Aboriginal (*n* = 109) and Torres Strait Islander (*n* = 1) parents aged >16 years by phone to complete the ACTSQ. Underlying domain structures were investigated with exploratory factor analysis and reviewed by experts to refine. Reliability and inter-rater reliability were assessed using McDonald’s Omega and Intraclass Correlation Coefficient (ICC).

**Results:**

Forty-four items on five factors were retained. Factors were labelled complex trauma symptoms (16 items), grief, loss and disconnection (6 items), support and relationships (9 items), sense of self and strengths (7 items), and Cultural connections and resources (6 items). There were moderate correlations between factors, with the exception of factor 5. Omega was >0.75 for all factors. The inter-rater reliability for each factor was fair to good (ICC 0.5–0.7).

**Conclusions:**

This study conducted a comprehensive psychometric validation that provides initial evidence towards the cultural validity of the ACTSQ to support assessment of complex trauma and strengths among Aboriginal and Torres Strait Islander peoples. Future studies are required to replicate and further evaluate the psychometric properties of the ACTSQ using larger samples.

## KEY POINTS

**What is already known about this topic:**

1. Complex post-traumatic stress disorder (complex trauma) is formally recognised in the International Classification of Diseases Eleventh Revision (ICD-11) to describe a cluster of symptoms frequently associated with prolonged exposure to inescapable threats or abuse, and often involving interpersonal violation.
2. For Aboriginal and Torres Strait Islander peoples in Australia impacted by complex trauma, there may be compounding factors, such as experiences of by historical trauma, loss and socio-economic deprivation stemming from colonisation.
3. Tools to assess complex trauma have been validated in several populations, but not among Aboriginal and Torres Strait islander peoples.

**What this topic adds:**

1. This paper presents the psychometric evaluation of a questionnaire designed to measure complex trauma and strengths in Aboriginal and Torres Strait Islander parents, the Aboriginal and Torres Strait Islander Complex Trauma and Strengths Questionnaire (ACTSQ). Aboriginal and Torres Strait Islander parents (*n* = 110) from three Australian jurisdictions were included in an exploratory factor analysis.
2. The ACTSQ provides culturally validated items across six complex trauma domains. Items that are aligned with symptom clusters identified in the International Trauma Questionnaire are included. In addition, through a rigorous process of Aboriginal-led community co-design, three other domains are identified as important: grief, loss and disconnection; strengths and sense of self; and sense of belonging.
3. The ACTSQ provides culturally validated items for the assessment of complex trauma-related distress and strengths among Aboriginal and Torres Strait Islander families. Field testing and refinement is now required.

## Introduction

Complex post-traumatic stress disorder (complex trauma) is formally recognised in the International Classification of Diseases Eleventh Revision (ICD-11) to describe a cluster of symptoms frequently associated with prolonged exposure to inescapable threats or abuse, often involving interpersonal violation (World Health Organisation, 2019). These symptom clusters include “affect/emotional dysregulation”, “negative self-concept”, and “relational disturbances”, in addition to previously recognised “post-traumatic stress disorder” symptom clusters (re-experiencing, avoidance and hypervigilance) (World Health Organisation, 2019). “Adverse childhood experiences” (ACEs) (Felitti et al., 1998) are common across all populations (Cloitre et al., 2019). Some populations are more at risk of experiencing ACEs and their sequelae due to social and economic conditions (Giano et al., 2020). Increasing evidence demonstrates ACEs are associated with wide ranging physical, social and emotional health problems throughout life (Felitti et al., 1998; Font & Maguire-Jack, 2016; Hughes et al., 2017). Critically, these effects may be exacerbated during the important life course transition to becoming a parent (Chamberlain, Ralph, et al., 2019), leading to compounding intergenerational cycles of trauma (Yehuda & Lehrner, 2018). Hence, addressing trauma at all stages of life is an international public health priority for improving health equity (Sara & Lappin, 2017; van der Kolk, 2000).

Tools to help identify people experiencing complex trauma are needed to understand the extent of the problem and strengthen pathways to support (Nestgaard Rød & Schmidt, 2021). The International Complex Trauma Questionnaire has been developed (Cloitre et al., 2018) and validated to measure complex trauma in a growing number of populations (Christen et al., 2021; Cloitre et al., 2019). Validation of tools in specific contexts is important because psychological constructs are context-based and shaped by cultural beliefs, including experiences and understandings related to how individuals interpret and respond to adversity and distress. This is particularly critical for complex trauma which represents a constellation of symptoms that most frequently manifest as a result of victimisation that occurs within complex environments of adversity involving multi-systemic influences (Leuchter et al., 2021). These systemic or structural level influences include wide-spread human rights violations and prolonged discrimination over generations.

Aboriginal and Torres Strait Islander peoples in Australia are one of the longest continuing cultures on earth, with over 250 distinct Countries and languages. Available evidence suggests that prior to colonisation, Aboriginal and Torres Strait Islander peoples were generally healthier than Europeans (Jackson & Ward, 1999). This state of health was not achieved by accident. Sophisticated and comprehensive lores and practices to foster wellbeing from before birth until after death were informed by understandings of connectedness as being central to social and emotional wellbeing (Gee et al., 2014), and fostering safety and nurturing individual agency from early childhood (McMahon, 2017). The centrality of connectedness to culture and kinship networks may be a particularly important consideration within the context of complex trauma, given antecedent traumatic experiences are often associated with interpersonal violation (World Health Organisation, 2019). From the earliest waves of colonisation, Aboriginal and Torres Strait Islander children, adults and whole families and communities suffered “severe inescapable threats”. These included violence, rape, dispossession of land, discrimination, severe socio-emotional deprivation and other human rights abuses (J. Atkinson, 1990), and the removal of children from families, an experience still occurring in the present time (Chamberlain, Gray, Bennet, et al., 2022). Concurrently, Aboriginal and Torres Strait Islander knowledge and cultural systems were intentionally denied, with punishments imposed for using cultural practices and language; and government control over every aspect of Aboriginal and Torres Strait Islander people’s lives. As a whole, the health of Aboriginal and Torres Strait Islander people has gone from being among the best to the worst in the world in just two hundred years (Anderson et al., 2016). National efforts to improve health equity over the 15 years have had limited success (Milroy & Bandler, 2021).

The resilience and strengths of Aboriginal and Torres Strait Islander communities have been demonstrated in survival, resistance to assimilation, and more recently cultural revitalisation. Yet the net effect of colonisation for many families is a legacy of compounding cycles of intergenerational trauma (J. Atkinson et al., 2014) and complex trauma (Clark et al., 2020) that are likely to be driving persistent health inequities (Chamberlain, Gee, et al., 2019). Research and knowledge, generated predominantly by non-indigenous people about Aboriginal and Torres Strait Islander peoples, too often focusses on failure and dysfunction resulting in a “deficit discourse” that does not reflect the strengths and resilience of Aboriginal and Torres Strait Islander peoples (Fogarty et al., 2018). These unique contextual factors reinforce the need for tools to include assessment of complex trauma-related distress and strengths that are culturally grounded and validated by Aboriginal and Torres Strait Islander peoples (Mushquash & Bova, 2007).

The transition to parenting is an important time: complex trauma-related distress can be exacerbated during this period, yet at the same time this period can also offer an opportunity for potential recovery for parents and prevention of negative impacts on the next generation (Chamberlain, Gee, et al., 2019; Marmot et al., 2012). Healing the Past by Nurturing the Future (HPNF) is a formative Aboriginal-led, community-based participatory action research (action research) project, which aims to co-design resources to improve perinatal care for Aboriginal and Torres Strait Islander parents across four key domains (Chamberlain, Gee, et al., 2019):

1. Awareness among service providers and parents of the impact of complex trauma or “trauma-informed” perinatal care to minimise the risks of triggering and compounding trauma responses (Fiolet et al., 2022).
2. Safe recognition of parents who may benefit from assessment and support, with processes to reduce risk of harm (Chamberlain, Gray, Herrman, et al., 2022).
3. Assessment of the experience of complex trauma to accurately identify parents living with complex trauma-related distress (Chamberlain et al., 2020).
4. Support strategies for parents, including psychological/emotional, social, cultural, and physical strategies (Chamberlain et al., 2021; Clark et al., 2022; Reid et al., 2021, 2022).

Addressing domain 3, this sub-study aimed to develop a valid tool for parents that assesses key dimensions of complex trauma aligned with Aboriginal and Torres Strait Islander perspectives (Chamberlain et al., 2020). The focus on parents was underpinned by the understanding that development of a tool to help identify parents experiencing complex-trauma related distress may help to support their mental health and social and emotional wellbeing, and potentially strengthen parenting practices. Community input during co-design called for the inclusion and consideration of strengths in addition to ascertaining complex trauma symptoms, hence this tool is called the Aboriginal and Torres Strait Islander Trauma and Strengths Questionnaire (ACTSQ).

This paper provides the first evaluation of the psychometric properties of the ACTSQ and discusses the lessons learnt that will inform the next phase of the work.

## Methods

### Preceding co-design work and questionnaire development

**Figure 1.**
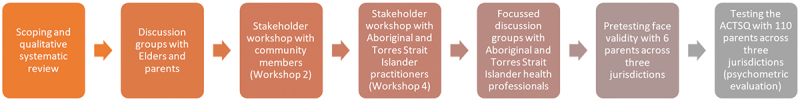
Summary of steps involved in development and testing the ACTSQ.

This study was informed by co-design work conducted within Intervention Mapping and Action Research cycles during a series of workshops from 2018 to 2020 (Figure 1, Appendix A). The Indigenous research methodologies and mixed method used are detailed elsewhere (Chamberlain, Gee, et al., 2019).

The preliminary list of potential ACTSQ items was developed using a multi-stage approach with input from key stakeholders. As shown in Figure 1 and Appendix A, this included a scoping and qualitative systematic review, discussion groups with Elders and parents, a stakeholder workshop with community members (Workshop 2), Aboriginal and Torres Strait Islander practitioners (i.e., psychologists, social workers) and academics (Workshop 3), discussion groups with Aboriginal and Torres Strait Islander health professionals, consultation with key experts, and pretesting face validity with six parents across three jurisdictions (Vic, SA and NT) (Chamberlain et al., 2020). Development details will be described further in a separate publication.

The preliminary list of ACTSQ items comprised 88 questions with a five-point Likert scale (0–4). The five-point Likert scale response categories included “not at all”, “a little bit”, “somewhat”, “a fair bit”, and “a lot”. Participants also had an option to respond “prefer not to say”. Participants with a response of “prefer not to say” for any item were excluded from the analysis. The preliminary ACTSQ included 28 strengths and 60 distress items. development process, these items had been categorised into 10 theoretical domains, which were drawn from the literature and workshops and pretested with parents and interviewers. These initial theoretical domains included: cultural and community connections; cultural disconnections; re-living the past and feeling fearful; relationships; relationships (parenting); relationship difficulties; grief and loss and spirituality; positive self and managing feelings; negative self and managing feelings; and self-harm. Following pre-testing feedback from parents and interviews, the original 88 items were not divided according to the 10 theoretical domains in the formatted structure of the ACTSQ. A full list of items is available on request. Following the ACTSQ development, the next stage was the psychometric evaluation, aiming to reduce the long list of items to a shorter list, which is the focus of this paper.

### Psychometric evaluation of the ACTSQ

#### Study design

This psychometric evaluation was a cross-sectional study, conducted in three Australian jurisdictions, selected on the basis of existing research relationships and interest expressed by key stakeholders in Northern Territory (NT), South Australia (SA), and Victoria (Vic). Data collection took place between October 2020 and May 2022. Data collection was originally scheduled to commence in April 2020, but was delayed due to a global coronavirus (COVID-19) pandemic, declared in March 2020. Adaptions included adding additional COVID-19 questions to evaluate effects of the pandemic (manuscript in progress), pivoting from face to face to online or phone interviews, and additional safety precautions to ensure parent wellbeing. The pandemic is unlikely to impact on the psychometric analysis presented in this paper, but may impact the prevalence of trauma-related distress. Interviews were conducted over the phone and data entered directly into a bespoke REDCap form (Harris et al., 2009).

#### Participants

Participants were initially eligible to take part if they: self-identified as Aboriginal and/or Torres Strait Islander; were aged over 16 years; were an expectant parent or a parent of a child aged under five. Parents were excluded if they were: under 16 years of age; aged 16 or 17 years and not considered sufficiently mature to understand and provide informed consent or were not willing to ask their own parents or guardians to provide parental consent for them to participate. Due to recruitment challenges, after 7 months the inclusion criteria were expanded to include parents of children of any age in May 2021. Participants were given a gift voucher for $35 for each interview completed, a children’s book, and a small gift.

### Ethical considerations

We acknowledge past harms from poorly conducted research with Aboriginal and Torres Strait communities and the potential risks of research involving Aboriginal and Torres Strait Islander parents experiencing complex trauma (Chamberlain, Gray, Herrman, et al., 2022). Hence, Human Research Ethic Committee (HREC) applications for the HPNF co-design research activities were submitted in three “phases” (see Appendix A) to ensure action learning and reflection from previous phases could be incorporated into the third “phase” submission, which included psychometric evaluation of the proposed assessment tool items. Phase three ethics approvals were granted from ethics committees in Victoria (St Vincent’s Hospital HREC 060/20; DHHS, La Trobe University; Royal Women’s Hospital; University of Melbourne), South Australia (AHREC 04-20-873), Women’s and Children’s Health Network) and Northern Territory (Central Australian HREC CA-20-3705). All participants were required to sign a Participant Information and Consent Form (PICF) prior to taking part. Participants had the opportunity to ask site staff and/or the research team any questions and discuss any concerns prior to signing the PICF.

#### Sample size

The study protocol (Chamberlain, Gee, et al., 2019) determined that a sample size target of 173 (approximately 55–60 in each of the three jurisdictions) Aboriginal and/or Torres Strait Islander parents would be required to assess the sensitivity and specificity of the ACTSQ compared to a gold standard (the ITQ). This was based on estimated prevalence of post-traumatic stress disorder (PTSD) of 20% reported in previous studies (C. L. Atkinson, 2008; Ben‐Ezra et al., 2018; Chamberlain, Gee, et al., 2019; Gartland et al., 2016; Hyland et al., 2017; Muzik et al., 2016). While the original research protocol and sample size calculation included comparing the sensitivity and specificity of the ACTSQ with the ITQ, this paper focuses only on exploring the factor structure of the ACTSQ. A future paper will discuss the sensitivity and specificity of the ACTSQ in comparison to the ITQ. In addition, future work will involve utilising a larger sample size to replicate and extend this preliminary analysis. This is consistent with the understanding that whilst there are no universal guidelines for sample size in exploratory factor analysis, broadly the psychometric literature recommends larger numbers (e.g., *n* = 300) for greater computational power (e.g. Floyd & Widaman, 1995; Guttman, 1945).

#### Recruitment

A range of recruitment methods was used. These were adjusted, as recruitment coincided with the onset of the COVID-19 global pandemic. Firstly, flyers were distributed in waiting rooms of partner perinatal health services to parents and expectant parents receiving routine care. Participating sites provided study information to parents who then contacted the researchers if they were interested in participating. There was also the option for site staff to record parents’ consent to be contacted by study staff directly. Secondly, participants were recruited through community networks, that included maternal, perinatal, community health and hospital services by email, social media and newsletters. Paid social media was also trialled using Facebook but was not effective in recruiting additional participants.

### Measures

All participants completed the preliminary ACTSQ items. Open-ended questions were included at the beginning and end of each section of the questionnaire to facilitate engagement, safety, and “checking in” about wellbeing. We also provided an opportunity for feedback from the participants about the questions (e.g., ease of understanding, difficulty answering). To assess inter-rater reliability, approximately 10% of participants were asked to complete the ASCTQ twice, with a different interviewer. These participants were randomly allocated.

During the interviews, participants were also provided basic sociodemographic information and completed the ICD-11 Trauma Questionnaire (ITQ) and a set of questions about COVID-19 experiences (Kennedy et al., 2022). This paper focusses on the ACTSQ only.

#### Interviewer training

Given the sensitive nature of the items, we planned for trained interviewers with experience in working safely with Aboriginal and Torres Strait Islander families to support participants to complete the questionnaire, either in person, by phone or Microsoft Teams. Interviewers were qualified health professionals or researchers. All interviewers underwent structured interviewer training which involved 4 hours of self-learning, then one day training with senior investigators, followed up by supervision by a clinical psychologist. Ongoing peer support meetings facilitated by senior investigators and a mental health professional were also provided. To help build cultural safety, participants were asked to provide their preferences for interviewers, with respect to gender and Aboriginal or non-Aboriginal status. Initially, the research team had planned to give participants the option of completing the questions themselves on a tablet, with support from an interviewer. However, with pivoting to online interviews due to the COVID pandemic, all participants were interviewed, and all questionnaires were completed by the interviewers. All interviewers used the REDCap form to record participant responses.

#### Ensuring safety and managing distress

Given that this project focuses on complex trauma, there was a recognised risk that participants and interviewers could experience distress or discomfort. A detailed cultural and emotional safety framework (Clark et al., 2020) and operational manual were developed for the project and followed by the research team. A flowchart for responding to distress is detailed in Appendix B, and interviewers were trained to follow this should participants become upset or distressed during the interview. Participants were referred to existing support services and other relevant services as required. They were also provided with a Thank You and Support Booklet after the interviews with a list of self-referral and support options. In addition, a wellbeing check was conducted with the participant approximately 1 week after the interviews. This included asking for feedback on the process, which resulted in a minor change to “onboarding” steps in the first month. The minor change to the “onboarding” process involved moving the COVID questions for participants to the first engagement point with a HPNF interviewer. Hence, participants answered related COVID questions during the pre-tool completion interview, rather than during the ACTSQ interview.

#### Data storage and analysis

All data were securely stored using REDCap software and accessible only to members of the project team (Harris et al., 2009). Data analysis was conducted using Stata SE version 17.0 (StataCorp, 2021).

Descriptive statistics were used to summarise the demographic characteristics of the sample, using frequencies and means (e.g., parent/child gender, parent/child age, Australian state). Analyses were stratified by state (Vic, SA or NT), parent gender, number of children (0, 1–2, 3+), parent age group (<20, 20–29, 30+ years), partner status, financial security (Health Care Card), educational level, and postcode.

#### Factor analysis

The underlying structure of the items was investigated with an exploratory factor analysis of a polychoric correlation matrix with listwise deletion. Any participant responses with “prefer not to answer” were set to missing and then excluded from analysis. The number of factors to retain was informed by visual inspection of a scree plot and running a parallel analysis (Dinno, 2009). We used the principal factor extraction method followed by promax rotation, given the likelihood of factor intercorrelation. This initial step guided subsequent decision-making with the research team about removing lower loading items to generate a shorter item list for this questionnaire. Given the preliminary developmental design stage of the measure, this decision making was also informed by Aboriginal clinicians and practitioners reviewing the factors for conceptual clarity with regard to therapeutic utility. The number of factors and distribution of items were reviewed with two key considerations from the Aboriginal clinicians and practitioners. This included, from a practice wisdom perspective: (1) did the factors reviewed make conceptual sense with regard to applying them in an assessment context that was congruent with an Aboriginal and Torres Strait Islander mental health and social and emotional wellbeing framework; and (2) did the result lend itself to an assessment of complex trauma that included both complex post-traumatic stress disorder and cultural idioms of distress, that was parsimonious, logical, and had practical utility and feasibility.

#### Item selection

The selection of items to retain in this questionnaire was guided by a combination of statistical indices, clinical, theoretical and cultural considerations. In addition to the factor analysis, qualitative data obtained through the open-ended questions on the ACTSQ was analysed using thematic analysis. Finally, given limitations in the sample size, and the large number of items included in the factor analysis, additional feedback was sought from members of the original working group who developed the potential ACTSQ items in a series of three meetings. The working group was comprised of several Aboriginal practitioners (i.e., psychologists, social workers, midwives, family support workers, doctors), and Aboriginal and non-Aboriginal academics who were investigators on the grant (included authors CA, CC, YC, SA, AE, GG). For this initial analysis, meeting participants agreed that it was important to be overinclusive, so that the tool could be refined after further field testing. The meeting participants considered the following to determine which items would be retained and which would be removed:

- Item performance on factor analysis (i.e., factor loadings > 0.3 with minimal cross loading).
- Qualitative feedback from participants regarding clinical and cultural clarity.
- Item content duplication or similarity to other items within this questionnaire or other tools routinely used in perinatal assessment (e.g., Edinburgh Postnatal Depression Scale (Cox et al., 1987), Drug and Alcohol Use). In choosing between duplicated items, preference was given to strengths-based items over distress-based items as per community feedback in the co-design.

The working group considered the content of items loading on each factor to derive labels for the construct being measured. Consensus was reached through discussion. Items were considered with regards to: qualitative feedback from participants about any challenges understanding items, consideration of duplication of items with preference for strength-based language as per co-design feedback from communities and expert consultation regarding conceptual clarity and congruence with the factor and therapeutic utility. Using a step-wise systematic process, each item was reviewed with 44 items excluded for the following primary reasons: Challenges with language (*n* = 7); Duplication (*n* = 13); Low/lower factor loading (*n* = 11); Expert consultation re-conceptual clarity (*n* = 4); Group decision to exclude depression items similar to those already used in other scales (*n* = 4); and the need to modify items that asked whether specific symptoms of distress impacted participant functioning, so that the impact questions were aligned with the new factors (*n* = 5). Following the meeting and expert consultation (HM), a second factor analysis was conducted with the items selected for retention to provide a final check of the factor structure. Internal consistency reliability coefficients (McDonald’s Omega) were computed with values 0.8 or above considered acceptable (Bland & Altman, 1997). Scales derived from each factor were generated by summing items, prorating for a maximum of one missing item per factor. We used prorating to maximise the sample size available for analysis. This method accounts for one missing item per subscale and does not reduce the subscale with data. It does this by first calculating the mean across the items (for participants with ≤1 missing response) and then multiplying that by the number of items per subscale. Items were reverse scored as necessary. To test convergent validity, associations between ACTSQ subscale scores and ITQ subscale scores were examined with Spearman’s correlation coefficient.

#### Inter-rater reliability

Inter-rater reliability was evaluated using an Intraclass Correlation Coefficient (ICC). Guidelines for interpreting agreement based on the ICC are varied and the clinical context should be considered. According to Cicchetti (1994): <0.40 is poor, 0.40–0.59 is fair, 0.60–0.74 is good, 0.75–1.00 is excellent (Cicchetti, 1994). According to (Bujang & Baharum, 2017), a minimum sample size of 13 participants gives 90% power to detect an ICC = 0.7 at two timepoints (assuming ICC = 0 in the null hypothesis). In addition, we calculated Pearson’s r and a paired t-test to assess parallel agreement.

## Results

The sample included 110 participants. Most participants identified as Aboriginal only (96%), 89% of the participants were female and their mean age was 33.9 years (see Table 1).

**Table 1.** Demographic characteristics of participating parents (Kennedy et al. 2022).

Demographics	*N* = 110 (%)
State	
South Australia	49 (44.6)
Victoria	52 (47.3)
Northern Territory	9 (8.2)
Gender	
Female	98 (89.1)
Male	11 (10.0)
Prefer not to say	1 (0.9)
Age, years, range	18–72
Age, years, mean (standard deviation)	33.9 (10.2)
Indigenous status	
Aboriginal	106 (96.4)
Torres Strait Islander	1 (0.9)
Both	3 (2.7)
Relationship status*	
Single	28 (25.7)
Partnered, living together	64 (58.7)
Partnered, not living together	11 (10.1)
Separated/Divorced	6 (5.5)
Number of children living with you**	
0 (pregnant)	10 (9.1)
0 (children left home)	2 (1.8)
1–2	68 (61.8)
>3	27 (24.6)
Prefer not to say	3 (2.7)
Highest level of education	
Some secondary schooling	14 (12.7)
Completed year 12	7 (6.4)
Other post-school education	61 (55.5)
Completed university degree	28 (25.5)

*Missing data for one participant; **missing data for three participants.

### Factor analysis

Only 11 participants answered “prefer not to say” on one or more items. This indicates the items were broadly acceptable. This left 99 participants with complete responses to all items, for inclusion in the factor analysis.

The scree plot and parallel analysis are provided in Appendix C. Close examination of the 2, 3, 4, 5 and 6-factor solutions suggested retention of five factors. In this solution, the majority of factor loadings >0.3 and there were negligible cross-loadings (Appendix D). After expert review of content, 44 items were retained and 44 were excluded (Table 2). The factors were labelled *complex post-traumatic stress disorder symptoms (representing 3 PTSD and 2 C-PTSD symptom clusters* via 16 items), *grief, loss and disconnection* (6 items), *supports and relationships (includes final C-PTSD symptom cluster of relationship difficulties)* (9 items), *sense of self and strengths* (7 items), and Cultural connections and resources (6 items). Factor 1 contained the most items, eight associated with post-traumatic stress disorder and eight associated with complex post-traumatic stress disorder. Factor 1 accounted for 28.0% of the variance, factor 2 17.0%, factor 3 16.7%, factor 4 15.7%, and factor 5 9.6%. There were generally moderate correlations between these factors, with the exception of factor 5, which showed small to negligible correlations with the other factors. The proportion of responses per category for each ACTSQ item is shown in Appendix E.

**Table 2.** Rotated factor matrix (factor analysis with polychoric correlations) for five component solution for items retained (n items = 44), blank cells indicate factor loading below 0.4.

	Factor				
Item description	Complex PTSD symptoms	Grief, loss & disconnection	Supports & connections	Sense of self & strengths	Cultural connections and resources
**Factor 1 : Complex post-traumatic stress disorder symptoms (*n* = 16)**					
Felt as if you are reliving bad or hurtful things that have happened	0.83				
Had a memory come back that was so strong you lost yourself in that moment	0.89				
Had flashbacks of bad or hurtful things that have happened without meaning to	0.85				
Had the same bad dreams or nightmares over and over again	0.61				
Tried to avoid people, places or situations that remind you of bad or hurtful things that have happened	0.72				
Felt on guard or “on alert”	0.71				
Felt angry really easily for no reason	0.85				
Felt edgy, jumpy, frightened or nervous	0.80				
Felt like there is something wrong with you	0.46				
Felt shame from things that have happened to me	0.77				
Felt guilty for no reason	0.79				
Felt you are no good	0.75				
Felt strong emotions and had trouble managing them	0.55				
Felt numb or found it hard to feel	0.66				
Felt your feelings can be hurt very easily	0.69				
Felt cut off or distant from what’s going on around you	0.47				
**Factor 2: Grief, loss and disconnection (*n* = 6)**					
I am able to spend time on “country” or a special place that I have a connection with		−0.72			0.48
Felt like your connection to spirit or spirituality is weak		0.40			
Felt grief and loss because of losing cultural knowledge and practices		0.93			
Felt grief and loss of connection to country		0.91			
Felt like your spirit or spirituality has become weak because of bad or hurtful things that have happened		0.66			
Felt disconnected to your ancestors because of bad or hurtful things that have happened	0.41	0.61			
**Factor 3: Supports and relationships (*n* = 9)**					
I have role models my life that I can learn about parenting			0.81		
I have people who listen to me and believe in me		0.44	0.64		
I feel supported by my friends/mob			0.44		
I have family that love me even when I muck up			0.79		
I have family/mob who can help me with my child			0.76		
I have people in my life that I have close relationships with			0.73		
Found it hard to have close, trusting relationships with people			−0.48		
Felt really alone, even when you are with others			−0.52		
Felt disconnected from people close to you, even when you know they care for you			−0.43		
**Factor 4: Sense of self and strengths (*n* = 7)**					
I feel like I have a say or can make choices in my life				0.46	
I feel it’s ok to be a “good enough” parent, and don’t have to be “perfect”				0.48	
I can trust myself to make good choices				0.60	
I feel ok with myself as I am				0.74	
I have done things that I am proud of				0.61	
I can manage my emotions well, even in difficult situations				0.79	
I am able to have a laugh even when things are difficult				0.74	
**Factor 5: Cultural connections and resources (*n* = 6)**					
I feel like I belong in my community					0.71
I participate in cultural practices that give me peace (such as going out bush, ceremony, community cultural events)					0.74
Spirituality is a source of strength for me					0.75
I have strategies to deal with racism if it happens				0.42	0.47
I am able to maintain my Aboriginal or Torres Strait Islander identity, values and beliefs					0.80
Not felt connected to your community					−0.45
% variance accounted for	28.3	17.0	16.7	15.7	9.6
McDonald’s Omega	0.93	0.85	0.85	0.82	0.76
Mean score (SD)	22.4 (14.6)	8.0 (5.8)	26.0 (7.3)	21.1 (4.7)	17.1 (4.9)
Range (max possible score)	0–58 (64)	0–23 (24)	11–36 (36)	9–28 (28)	4–24 (24)

#### Preliminary examination of convergent and discriminant validity

Spearman’s correlation coefficients were used to assess the convergent and discriminant validity of the ACTSQ. As presented in Table 3, Factor 1 Complex post-traumatic stress disorder symptoms, and Factor 2 Grief, loss and disconnection both showed positive correlations with the ITQ PTSD and CPTSD subscales. Conversely, Factor 2 Supports and relationships and Factor 4 Sense of self and strengths both showed negative correlations with the ITQ PTSD and CPTSD subscales. Factor 5 Cultural connections and resources did not correlate with either ITQ subscales.

**Table 3.** Spearman’s correlation matrix.

	1	2	3	4	5	6
1 Factor 1 Complex post-traumatic stress disorder symptoms	–					
2 Factor 2 Grief, loss, and disconnection	0.46	–				
3 Factor 3 Supports and relationships	−0.57	−0.38	–			
4 Factor 4 Sense of self and strengths	−0.45	−0.33	0.50	–		
5 Factor 5 Cultural connections and resources	−0.09	−0.29	0.24	0.37	–	
6 ITQ – PTSD symptom cluster	0.75	0.26	−0.52	−0.24	0.03	–
7 ITQ – CPTSD symptom cluster	0.73	0.36	−0.52	−0.35	−0.02	0.68

The four factors that correlated with ITQ subscale scores, with the exception of Factor 5 (Cultural connections and resources), provide preliminary evidence for adequate convergent and discriminant validity. Internal consistency was >0.75 for all factors.

Mean scores, and the range for each factor are shown in Table 2.

#### Interrater reliability

A total of 14 participants had a repeat ACTSQ questionnaire completed with a different interviewer. The mean time between interviews was 13 days (SD 10, range 2–35). The ICC for each factor was fair to good, ranging between 0.5 and 0.7. Factors 4 and 5 had lower correlations than the other factors (fair) (Table 4).

**Table 4.** ICC for each factor.

Factor	ICC	95% CI
Complex PTSD symptoms	0.71	0.32, 0.90
Grief loss and disconnection	0.63	0.17, 0.87
Supports and relationships	0.74	0.37, 0.91
Sense of self and strengths	0.49	0.02, 0.80
Sense of belonging	0.53	0.01, 0.82

ICC = Intraclass Correlation Coefficient; CI = confidence interval; PTSD = post traumatic stress disorder.

## Discussion

This study describes the process of psychometric evaluation and factor analysis of items of the Aboriginal and Torres Strait Islander Complex Trauma and Strengths Questionnaire. The preliminary questionnaire includes five factors: (1) 3 PTSD and 2 C-PTSD symptom clusters, (2) grief, loss and disconnection (3) supports and relationships (including final C-PTSD symptom cluster of relationship difficulties), (4) sense of self and strengths, and (5) Cultural connections and resources. These items were carefully selected and refined through a rigorous process of co-design and exploratory factor analyses. They reflect key domains aligned with Aboriginal and Torres Strait Islander understandings of complex trauma impacting parents and were tested by 110 Aboriginal and Torres Strait Islander parents responding to questions from a trained healthcare professional with experience of working safely with Aboriginal and Torres Strait Islander families.

Our study found that items representing five of six symptom clusters of complex trauma as defined by the International Complex Trauma Questionnaire (ITQ), loaded onto one factor. However, one symptom cluster – disturbances in relationships – loaded onto another factor (3) with items related to supports and relationships. This is not surprising given the centrality of connectedness in Aboriginal and Torres Strait Islander understandings of social and emotional wellbeing. This tool includes more nuanced categories related to connections and relationships (Gee et al., 2014), which was reinforced by parents in other components of the broader HPNF project (Chamberlain et al., 2021). Many of the included items were drawn from previous Aboriginal community research which formed the PhDs of Aboriginal authors (CA) on the Australian Aboriginal Version of the Harvard Trauma Questionnaire (C. L. Atkinson, 2008) and (GG) on the Aboriginal Resilience and Recovery Questionnaire (ARRQ: Gee, 2016; Gee et al., 2023). We note here that similar strength-based items from the ARRQ have been successfully used in other studies to assess cultural determinants of wellbeing (Gee et al., 2023). However, our study differs from previous studies of trauma-related distress, in identifying potentially important constructs of grief, loss and disconnection, sense of self and strengths, and cultural connections and resources, which impact on psychological wellbeing and are related to complex trauma. Items to identify these constructs can help to identify potential areas for clinical and practical support to address feelings of grief and loss, fostering reconnections, sense of belonging and personal reflective capacity about healing and recovery. Items within these additional constructs were identified and developed within the cultural context of one of the longest continuous living cultures on earth, which enjoyed comparative good health for millennia underpinned by sophisticated understandings of social and emotional wellbeing; up until relatively recent traumatic violence and oppression associated with colonisation. These findings offer rich insights grounded in Aboriginal and Torres Strait Islander culture and may well be important constructs for other communities elsewhere.

This tool was rigorously developed through 4 years of Aboriginal-led co-design. However, at this early stage of tool development, we acknowledge there are several important limitations that need to be acknowledged and addressed in follow-up studies. For example, we note that four items retained within the five-factor solution had cross loadings with a second factor. The four items are: “I am able to spend time on ‘country’ or a special place that I have a connection”, “Felt disconnected to your ancestors because of bad or hurtful things that have happened”, “I have people who listen to me and believe in me” and “I have strategies to deal with racism if it happens”. Conceptually this indicates that from a psychometric perspective, the item may be a poor representation of the indicated factor/construct. The expert consultation group carefully considered several issues with respect to the decision to retain these four items in the preliminary ACTSQ. First, each of these items represent key theoretical constructs related to the potential intersections and roles of culture (i.e., cultural practices and spirituality/ancestors), extended kinship networks as a support, and historical/social adversity (i.e., racism and grief and loss) in relation to trauma and healing. Second, to our knowledge, there is not a single First Nations study worldwide that is yet to investigate the psychometrically derived factor structure of constructs related to healing and recovery from trauma within the context of a complex trauma assessment tool. Therefore, due to the theoretical importance of the items, we believe it is premature to omit these items until we replicate this study using a larger sample size. The theoretical importance of these items is justified by several strands of evidence. For example, there are sound theoretical arguments for the central role of culture and context in influencing the relationship between trauma and resilience (e.g. Ungar, 2013), and recent empirical work has demonstrated that ethnicity/cultural affiliation may moderate the role between appraisals and trauma symptom severity (e.g. Bernardi & Jobson, 2019), Within an Aboriginal and Torres Strait Islander research context, the work of C. L. Atkinson (2008) and Gee (2023), both co-authors on this paper found relationships between trauma-related distress and cultural determinants. However, neither author used exploratory factor analysis as a method to investigate complex trauma and strengths-related constructs in a single assessment tool. On this basis, we believe it is warranted to retain these four items at such an early stage of theory and psychometric development. However, if future studies using a larger sample size with more computational power replicate these cross-loadings, then these items will be omitted.

We acknowledge that this study utilised a modest sample size of 110 parents rather than the target of 173, due to the impact of the COVID-19 pandemic. A sample size of 110 is less than what is generally recommended for an exploratory factor analysis that will replicate in other samples (Goretzko et al., 2021). Hence, we have erred towards inclusivity with this current list of items and will further investigate the factor structure, feasibility of reducing the items, item floor/ceiling effects, the sensitivity and specificity against the ITQ, the impact of the COVID-19 pandemic, and test–retest reliability over the perinatal period in subsequent field testing. It is particularly important to assess test–retest reliability, given the wide confidence intervals around the ICC, caused by the small sample size (*n* = 14). The test–retest reliability will be reassessed in future studies. This is important because becoming a parent is a transitional time, so it is likely there may be variability in responses even over a short period of time, influenced by a wide range of variable individual, relational and social factors and stage of recovery (Seng et al., 2002). We note that few of the parenting-specific questions that were developed specifically for this tool were retained as the items did not load highly onto any factor. This may have been a result of including too few parenting items in the item development phase to form a distinct factor. Hence, the final version includes items which are relevant for parents but not specific to their parenting. The Tool to Measure Parenting Self-Efficacy (TOPSE) is undergoing adaptation for use by Aboriginal and Torres Strait Islander communities at the time of drafting this manuscript and may be a useful adjunct to the ACTSQ for collecting data on parenting specific items. Third, this tool was developed with and for Aboriginal and Torres Strait Islander parents. Validation with other population groups is required to assess whether it is applicable, and its use can be extended.

We also note here the interesting finding that factors one through four representing complex trauma symptom severity, grief, and loss, supports and relationships, and personal sense of self and strengths, were all significantly associated with the ITQ PTSD and CPTSD symptom clusters, indicating convergent and discriminant validity. However, the cultural connection and resources factor was not. Whilst putting aside the already acknowledged design limitations of this study, such as the lack of power due to sample size – this finding raises questions about the relationship between constructs of culture or cultural determinants and post-trauma related distress. This is a new field of enquiry. While the importance of culture more broadly with regard to First Nations health and wellbeing is clearly established, the relationship between constructs of culture and specific patterns of distress remains limited, and early findings suggest it may not be straight forward. For example, the early PhD work of co-author Gee (2016) found that while a sense of opportunity in community and communal mastery were associated with lower post-trauma symptom severity among 81 help-seeking Aboriginal participants, cultural constructs such as cultural identity, spirituality and cultural practices were not. However, cultural practices were associated with high levels of self-reported empowerment among the participants. This suggests that constructs related to culture (e.g., cultural connection, cultural practices) may operate in different ways – both with respect to different types of health outcomes such as positive wellbeing versus distress, and indeed different forms of distress. For example, cultural resilience constructs, such as enculturation, cultural attachment, and participation in traditional practices have been found to be negatively associated with substance and alcohol misuse and depression (e.g., Dockery, 2010; Whitbeck et al., 2002). However, other studies, such as that of Torres Stone and colleagues (Stone et al., 2006) found that cultural and spiritual practices predicted alcohol cessation among Native American and First Nation adults from reservations in the Upper Midwest of the United States, whereas strength of cultural identity did not.

### Implications for practice, policy and research

The preliminary ACTSQ provides information for Aboriginal and Torres Strait Islander parents and healthcare professionals to identify areas of trauma-related distress to guide therapeutic support. Understanding strengths and coping strategies may be helpful for parents and clinicians in providing appropriate support and referrals. Our team is developing a support framework based on related research to help identify culturally appropriate support across a broad range of relevant domains (Reid et al., 2022).

It is important for clinicians to remember this is a tool to facilitate safe discussions. Caution should be used applying any arbitrary “diagnostic” labels to individuals. In the Power-Threat-Meaning Framework, the British Psychological Society outlines evidence for a conceptual alternative to psychiatric classification and recognising the importance of context for understanding emotional distress or troubling behaviour (Johnstone et al., 2018). It emphasizes the role of power in people’s lives, including recognising coping or survival responses to “threat”, rather than assuming pathology: that is, considering “what has happened to you?” rather than “what is wrong with you?” (Johnstone et al., 2018). We argue these scales should be used in a way which is consistent with the Power Threat Meaning Framework, helping parents to understand their own story about “what has happened to them” and understand the impact that is having for them now, and may potentially have for their parenting.

It is important to note that any assessment has the potential for risks of harm. Identifying complex trauma carries significant risks for Aboriginal and Torres Strait Islander parents due to potential inappropriate involvement of child protection services (O’Donnell et al., 2019) and it is critical that culturally appropriate governance and processes are followed to ensure any benefits of identification outweigh potential harm (Dobrow et al., 2018). This highlights the importance of including strengths-based questions when talking with Aboriginal and Torres Strait Islander parents about trauma. Future studies could explore the role of strengths as protective factors. We have raised concerns about potential risks for Aboriginal and Torres Strait Islander parents in the current maternal and child health care system in Australia. We propose that before any assessment processes are offered, there needs to be awareness and safety (Fiolet et al., 2022), acceptable support available (Reid et al., 2021, 2022), and strategies to ensure safe recognition of complex trauma (Chamberlain, Gray, Herrman, et al., 2022), with skilled and effective professionals and systems in place. We have refined preliminary text and requirements for using the ACTSQ and will be making it available to people who have completed mandatory training to foster safe use.

Addressing compounding cycles of intergenerational trauma in the transition to parenting is a critical foundation to closing the gap in health equities between Aboriginal and Torres Strait Islander peoples and other Australians. Trauma-integrated care needs to be integral to culturally safe continuity-of-care models currently being implemented nationally, consistent with “Birthing on Country” principles (Australian College of Midwives, 2017). In addition to clinical utility, this tool could be used to assess progress on addressing drivers of health inequity, and in other settings such as family support and education to help accurately measure the extent and impact of complex trauma on Aboriginal and Torres Strait Islander wellbeing – which can then inform targeted interventions and appropriate resource allocation.

Future research involving larger numbers of participants will help to refine the current items to a shorter more user-friendly length, evaluate the sensitivity and specificity in detecting complex trauma in comparison with existing tools, and the test–retest reliability over the perinatal period. It may be possible to identify a small number of highly sensitive items (i.e., 2–4 items) that are more acceptable to parents and could replace the existing “exposure” items currently recommended as part of routine antenatal screening in the Antenatal Risk Questionnaire (i.e., past experiences of physical, sexual and emotional abuse) (Reilly et al., 2021). Feedback from our co-design discussions suggests these direct questions are not appropriate for some women (Chamberlain et al., 2020), pose potential risks as some services use this information to justify notification of the parent as “at risk” to Child Protection Services (CPS) (Chamberlain, Gray, Herrman, et al., 2022), is unlikely to be answered accurately which means it has very little clinical utility (Owens et al., 2022), and offers modest predictive value for current or future distress (van Roessel et al., 2021). The utility of the ACTSQ in other settings also needs to be assessed. We also believe that the additional items identified as important components of complex trauma by Aboriginal and Torres Strait Islander peoples (connectedness more broadly, grief and loss, sense of self/strengths, and sense of belonging) offer unique insights and are likely to be relevant for other populations elsewhere and should be considered if appropriate.

## Conclusions

The preliminary ACTSQ provides rigorously developed and culturally validated items for assessment of complex trauma and important related constructs (grief, loss and disconnection, sense of self and sense of belonging) among Aboriginal and Torres Strait Islander parents. Field testing and refinement is now required, with safety protocols to ensure the benefits outweigh any harms to Aboriginal and Torres Strait Islander families. Using the ACTSQ in clinical practice may lead to improvements in both practice and policy, through increased knowledge and awareness of strategies to transform cycles of intergenerational trauma and hurt to cycles of intergenerational nurturing and recovery. This is critical for improving health and health equity so that Aboriginal and Torres Strait Islander children grow up healthy, happy and strong.

## Appendix D. Rotated factor analysis/pattern matrix (factor analysis with polychoric correlations) for 5 component solution, shaded cells highlight the highest factor loading for each item, blank cells indicate factor loading <3 (n items = 88)

**Table:**

Item	Factor weights					Item	Factor weights				
	**1**	**2**	**3**	**4**	**5**		**1**	**2**	**3**	**4**	**5**
**14**	0.59				−0.46	34			0.71		
**15**	0.42					35			0.60	0.50	
**16**	0.57					36			0.40	0.38	0.34
**17**	0.62				−0.53	37			0.66		0.32
**18**	0.30					38			0.68		
**20**	0.86	−0.31				39			0.78	0.31	
**21**	0.91					**40**			0.56	0.47	
22	0.90	−0.36				**41**			0.40	0.35	0.28
23	0.86					**44**			0.38	0.34	
24	0.87					46			−0.55		
25	0.49					47			−0.65		
26	0.76					48	0.31		−0.57		
**27**	0.83					**49**	0.32		−0.50		
28	0.64					**50**			−0.45		
29	0.65					**69**			0.30		
30	0.69					**82**			0.25		
**31**	0.38					**3**				0.34	
**32**	0.66					6		0.32		0.60	0.36
**33**	0.47					7				0.52	
**51**	0.33					**12**			−0.38	0.43	−0.42
**52**	0.42		−0.39			42				0.41	
**54**	0.67					**45**				0.22	
**56**	0.74				0.31	64		−0.35		0.51	
**63**	0.46	0.36				65				0.69	
71	0.54					66				0.64	
**72**	0.72			−0.36		67				0.59	
73	0.64					68				0.64	0.00
74	0.61			−0.32		**70**				0.39	
75	0.64					1					0.67
76	0.60					4				0.40	0.62
77	0.66					5					0.64
78	0.63					8					0.68
79	0.45	0.33				**9**					0.55
**80**	0.44	0.44				**10**		0.40			−0.50
**81**	0.37					11		0.44			−0.51
**83**	0.63					**13**		0.37			−0.58
**84**	0.53										
**85**	0.34					**43**			0.31		−0.31
**86**	0.62			−0.33							
**87**	0.63			−0.43		**55**					0.37
**88**	0.56										
2		−0.58			0.50						
**19**	0.30	0.37									
**53**		0.34									
57		0.34									
58		0.88									
59		0.89			−0.31						
60	0.37	0.54									
61		0.58			−0.36						
**62**	0.33	0.40									
Total loading to factor							41	9	16	12	10
% variance accounted for							29.8	18.6	17.4	11.8	6.5
Factor intercorrelations											
With Factor 2							0.51				
With Factor 3							−0.47	−0.40			
With Factor 4							−0.26	−0.30	0.23		
With Factor 5							0.00	0.06	0.06	0.03	

## Appendix E. Proportion of item responses per category for ACTSQ items

**Table:**

#	Item	Not at all (%)	A little (%)	Some what (%)	A fair bit (%)	A lot (%)
**Factor 1**						
Felt as if you are reliving bad or hurtful things that have happened		44.0	23.9	14.7	13.8	3.7
Had a memory come back that was so strong you lost yourself in that moment		42.7	24.6	13.6	10.0	9.1
Had flashbacks of bad or hurtful things that have happened without meaning to		26.4	32.7	17.3	13.6	10.0
Had the same bad dreams or nightmares over and over again		55.5	18.2	11.8	7.3	7.3
Tried to avoid people, places or situations that remind you of bad or hurtful things that have happened		30.9	13.6	15.5	20.0	20.0
Felt on guard or ‘on alert’		25.5	20.0	18.2	10.9	25.5
Felt angry really easily for no reason		32.7	22.7	21.8	17.3	5.5
Felt edgy, jumpy, frightened or nervous		32.7	13.6	25.5	18.2	10.0
Felt like there is something wrong with you		25.7	30.3	17.4	17.4	9.2
Felt shame from things that have happened to me		33.6	26.4	18.2	14.6	7.3
Felt guilty for no reason		32.7	20.0	22.7	17.3	7.3
Felt you are no good		39.1	25.5	20.9	7.3	7.3
Felt strong emotions and had trouble managing them		27.5	23.9	27.5	12.8	8.3
Felt numb or found it hard to feel		48.2	25.5	16.4	5.5	4.6
Felt your feelings can be hurt very easily		24.6	21.8	29.1	10.9	13.6
Felt cut off or distant from what’s going on around you		30.9	24.6	27.3	10.0	7.3
**Factor 2**						
I am able to spend time on ‘country’ or a special place that I have a connection with		29.1	15.5	25.5	20.0	10.0
Felt like your connection to spirit or spirituality is weak		45.9	22.0	21.1	8.3	2.8
Felt grief and loss because of losing cultural knowledge and practices		26.6	24.8	23.9	13.8	11.0
Felt grief and loss of connection to country		26.4	23.6	20.9	19.1	10.0
Felt like your spirit or spirituality has become weak because of bad or hurtful things that have happened		38.5	27.5	22.0	8.3	3.7
Felt disconnected to your ancestors because of bad or hurtful things that have happened		54.1	17.4	9.2	11.0	8.3
**Factor 3**						
I have role models my life that I can learn about parenting		8.2	12.7	17.3	14.6	47.3
I have people who listen to me and believe in me		0.0	7.3	13.6	22.7	56.4
I feel supported by my friends/mob		2.7	6.4	12.7	25.5	52.7
I have family that love me even when I muck up		1.8	2.7	9.1	15.5	70.9
I have family/mob who can help me with my child		9.1	15.5	11.8	9.1	54.6
I have people in my life that I have close relationships with		0.0	10.0	9.1	13.6	67.3
Found it hard to have close, trusting relationships with people		15.5	18.2	16.4	22.7	27.3
Felt really alone, even when you are with others		9.1	18.2	23.6	24.6	24.6
Felt disconnected from people close to you, even when you know they care for you		7.3	20.9	25.5	20.0	26.4
**Factor 4**						
I feel like I have a say or can make choices in my life		0.0	1.8	10.9	34.6	52.7
I feel it’s ok to be a ‘good enough’ parent, and don’t have to be ‘perfect’		7.3	4.6	16.4	21.8	50.0
I can trust myself to make good choices		0.0	0.9	14.7	39.5	45.0
I feel ok with myself as I am		0.9	14.7	30.3	25.7	28.4
I have done things that I am proud of		0.9	3.6	17.3	28.2	50.0
I can manage my emotions well, even in difficult situations		3.6	12.7	34.6	29.1	20.0
I am able to have a laugh even when things are difficult		0.9	6.4	18.2	36.4	38.2
**Factor 5**						
I feel like I belong in my community		3.6	10.9	15.5	21.8	48.2
I participate in cultural practices that give me peace (such as going out bush, ceremony, community cultural events)		10.0	23.6	20.0	22.7	23.6
Spirituality is a source of strength for me		7.3	8.2	18.2	16.4	50.0
I have strategies to deal with racism if it happens		8.2	12.7	21.8	29.1	28.2
I am able to maintain my Aboriginal or Torres Strait Islander identity, values and beliefs		2.7	1.8	13.6	20.0	61.8
Not felt connected to your community		6.4	7.3	20.0	18.2	48.2
Reverse scored = 5, 49, 50, 51, 14						
